# Transverse Colon Volvulus Secondary to the Persistent Descending Mesocolon: A Case Report

**DOI:** 10.7759/cureus.61272

**Published:** 2024-05-28

**Authors:** Jumpei Shibata, Akihiro Tomida, Masaoki Hattori, Motoi Yoshihara

**Affiliations:** 1 Surgery, Nishichita General Hospital, Tokai, JPN

**Keywords:** surgical risk factors, case report, laparoscopic partial colectomy, persistent descending mesocolon, volvulus, transverse colon

## Abstract

This case report introduces a rare occurrence of transverse colon volvulus associated with persistent descending mesocolon (PDM), a congenital anomaly characterized by the medial positioning of the descending colon due to a failed fusion with the dorsal abdominal wall. We detail the case of an 18-year-old female, with a medical history of surgically corrected coarctation of the aorta and anal atresia, who presented with recurrent transverse colon volvulus despite having undergone a laparoscopic colopexy three years earlier. Physical examination revealed abdominal distension and metallic colic sounds while imaging studies confirmed the recurrence of the volvulus. Laparoscopic partial resection of the transverse colon was performed, which revealed a medially positioned descending colon due to PDM. Postoperative complications included anastomotic failure, necessitating a second operation. The patient was successfully discharged without further complications after seven days. This case underscores the clinical significance of recognizing PDM, highlighting its potential role in causing transverse colon volvulus and increasing the risk of anastomotic failure. It emphasizes the need for surgeons to remain vigilant regarding this congenital anomaly to mitigate unexpected outcomes such as recurrent volvulus and postoperative complications.

## Introduction

Persistent descending mesocolon (PDM) is a congenital condition characterized by the medial positioning of the descending colon due to the unsuccessful fusion with the dorsal abdominal wall. The descending and sigmoid colon undergo a rightward shift, adhering to the intestinal mesentery. First documented in 1960 [1], its incidence ranges from 1.3% to 4.0% [2-4]. While many cases remain asymptomatic, certain clinical complications, such as sigmoid colon volvulus and intestinal obstruction, have been documented [5]. Notably, recent studies have identified PDM as an independent risk factor for prolonged operative times and anastomotic failures in sigmoid and rectal cancer surgeries [6], gradually gaining recognition for its clinical significance.

Volvulus refers to the torsion of a segment of the alimentary tract, resulting in bowel obstruction and ischemia. The sigmoid colon and cecum are commonly affected sites (43％ and 52％, respectively) [7], with volvulus occurring in other alimentary tract portions, such as the transverse colon, being infrequent.

This report details a case of transverse colon volvulus attributed to PDM, aiming to increase awareness of this congenital anomaly and underscore its role as a potential risk factor in colon anastomosis.

## Case presentation

An 18-year-old female presented to our hospital with a history of repetitive transverse colon volvulus. Her medical background included coarctation of the aorta and anal atresia, both of which required surgical intervention. Despite her complex medical history, she did not exhibit mental retardation. Three years prior, she underwent laparoscopic colopexy to address the transverse colon volvulus, during which her transverse and descending colon were affixed to the left lateral peritoneal wall using absorbable sutures [8].

Upon physical examination, metallic colic sounds and abdominal distention were noted, with no evidence of rebound tenderness. The initial laboratory test revealed no significant abnormality (Table 1).

**Table 1:** Laboratory data MCV: mean corpuscular volume, AST: aspartate aminotransferase, ALT: alanine aminotransferase, ALP: alkaline phosphatase, γ-GTP: γ-glutamyl transpeptidase, BUN: blood urea nitrogen, CK: creatine kinase, CRP: C-reactive protein

Parameter	Result	Reference value
Peripheral blood		
White blood cells	6300	3300-8600 (/μL)
Red blood cells	473	370-500 (×10^4/μL)
Hemoglobin	13.4	11.0−15.0 (g/dL)
Hematocrit	43.1	35.0−48.0(％)
MCV	91.1	83-100 (fL)
Platelets	23.5	15-40 (×10^4/μL)
Neutrophils	56	35-70 (%)
Eosinophils	1	1-6 (%)
Basophils	0	0-1 (%)
Monocytes	6	3-10 (%)
Total protein	7.5	6.7-8.3 (g/dL)
Albumin	4.6	4.0-5.0 (g/dL)
AST	26	13-33 (IU/L)
ALT	24	6-27 (IU/L)
ALP	68	38-113 (U/L)
γ-GTP	12	10-47 (IU/L)
Total bilirubin	0.7	0.3-1.2 (mg/dL)
BUN	12.3	8-22 (mg/dL)
Creatinine	0.63	0.4-0.7 (mg/dL)
CK	56	45-163 (IU/L)
Na	140	138-146 (mEq/L)
K	4.2	3.6-4.9 (mEq/L)
Cl	102	99-109 (mEq/L)
Glucose	93	73-109 (mg/dL)
CRP	0.3	0-0.39 (mg/dL)

**Figure 1:**
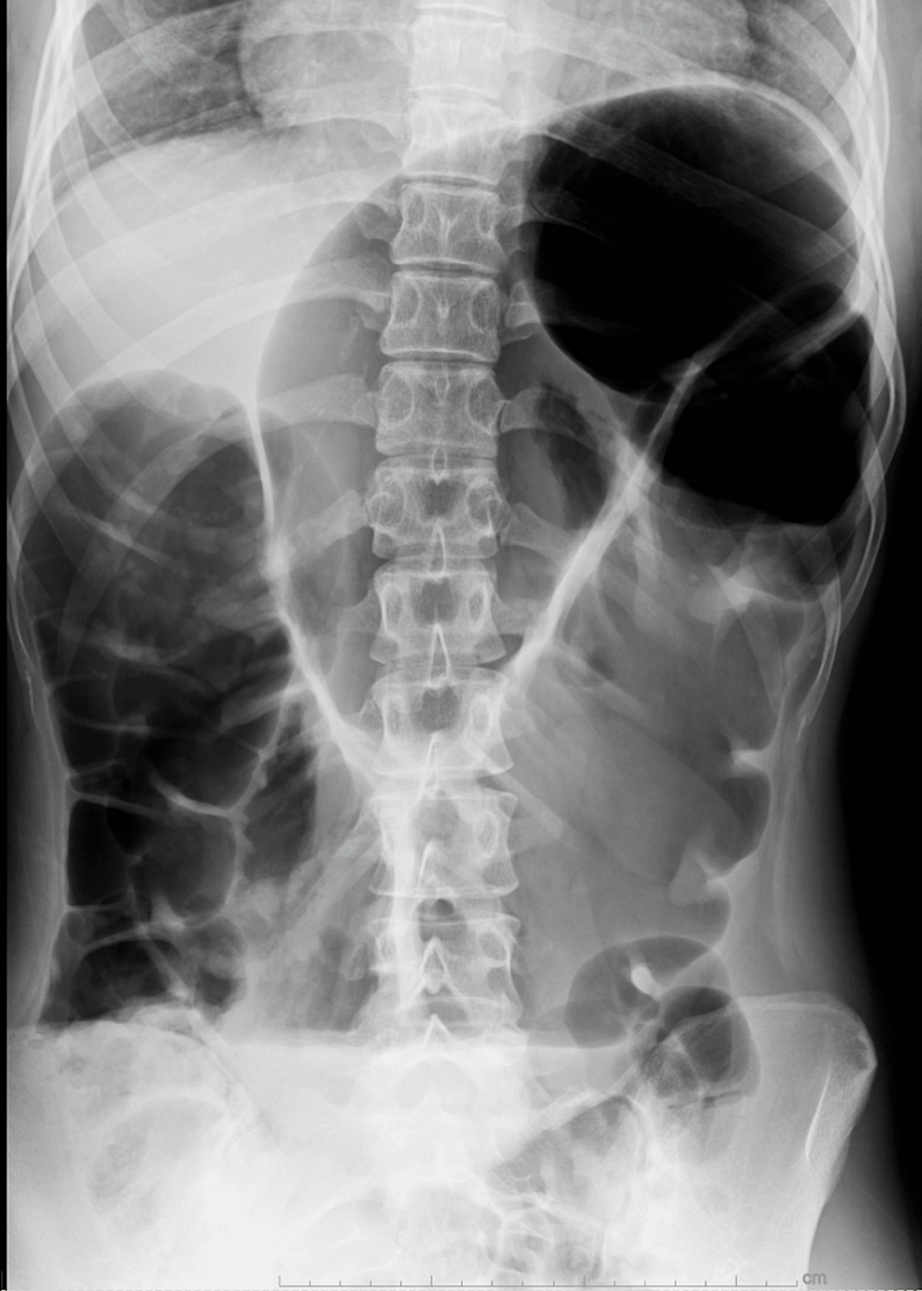
Plain abdominal radiography Plain abdominal radiograph demonstrating a notably distended ascending and transverse colon

A plain abdominal radiograph illustrated a distended transverse colon, known as the "bent inner tube" sign (Figure 1).

**Figure 2:**
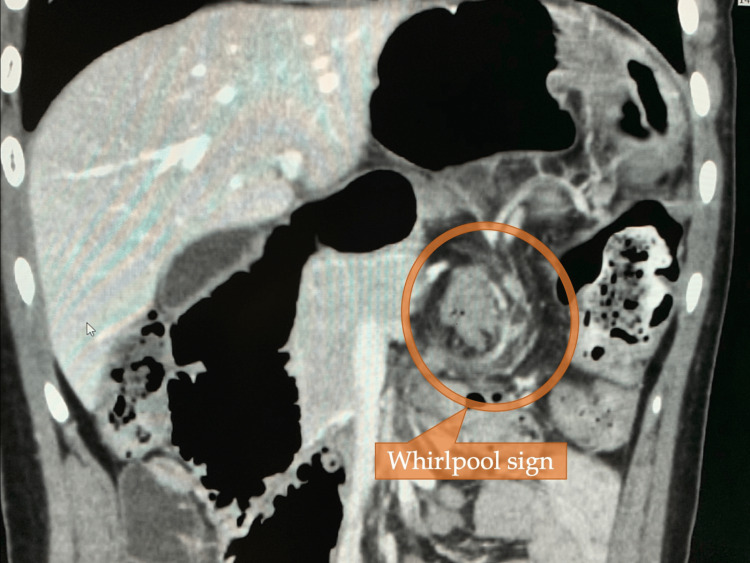
Contrast-enhanced CT scan showing a whirlpool sign The CT scan represents the whirlpool sign, the swirling appearance of the transverse colon mesentery.

**Figure 3:**
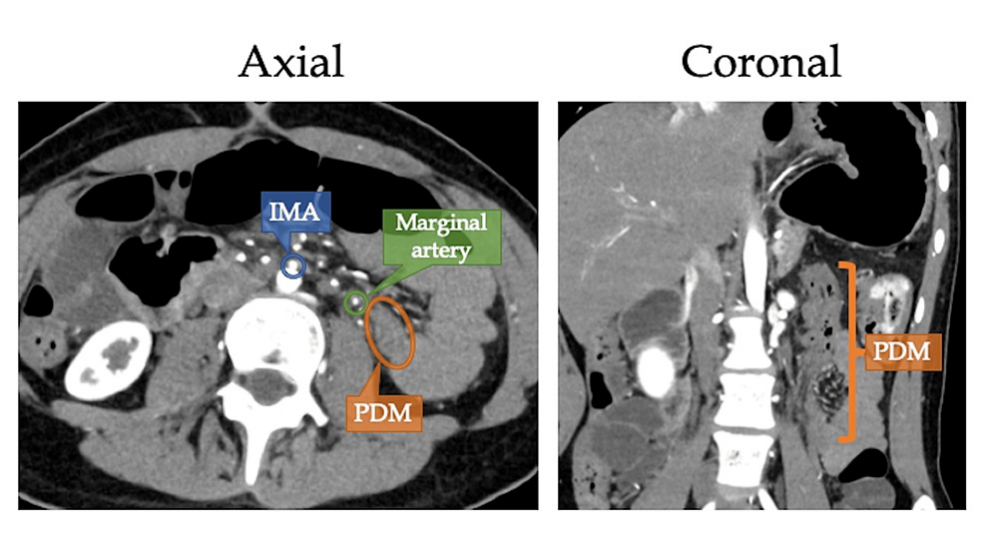
Contrast-enhanced CT scan representing PDM The CT scan showcases the dilation of the transverse colon, with the descending colon situated in the midline. The measurements, including the distance between the right border of the IMA and the right border of the marginal artery arch, and the right border of the IMA and the right border of the descending colon (21 mm and 24 mm, respectively), fall below the normal range (27 mm and 35 mm on average). PDM: persistent descending mesocolon; IMA: inferior mesenteric artery

Subsequent contrast-enhanced computed tomography (CT) unveiled the whirlpool sign in the transverse mesentery (Figure 2) along with the medialization and oblique course of the descending colon features characteristic of persistent descending mesocolon (PDM). Measurements indicated distances of 21 mm between the right border of the inferior mesenteric artery (IMA) and the right border of the colonic marginal artery arch, and 24 mm between the right border of the IMA and the right border of the descending colon (Figure 3).

**Figure 4:**
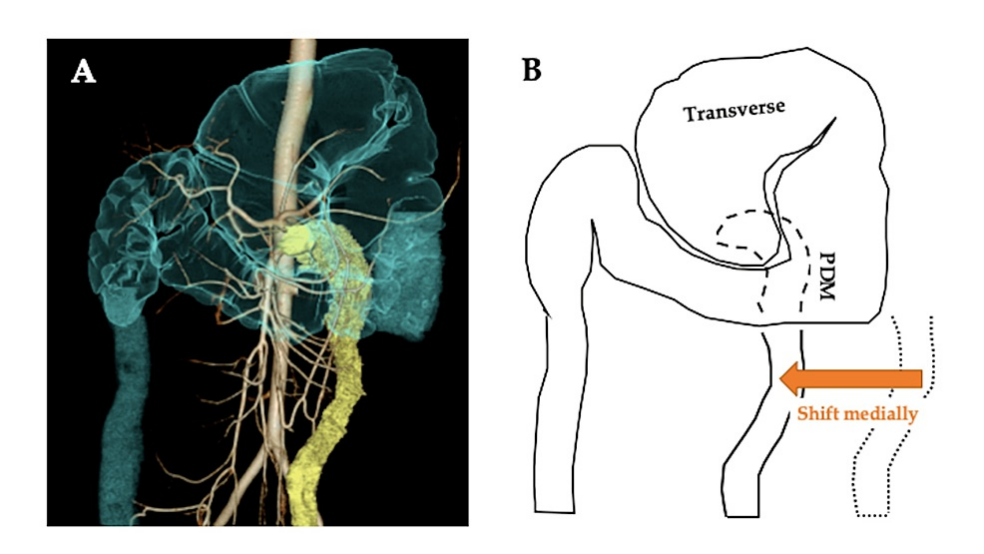
Three-dimensional CT imaging A) Depiction of the twisted ascending and transverse colon in blue, with PDM highlighted in yellow, running along the medial side. B) Schematic representation illustrating the redundancy of the transverse colon and its mesentery due to the midline shift of the descending colon. PDM: persistent descending mesocolon

Three-dimensional CT imaging revealed the twisted colon's root to be the spiral upper-descending colon (Figure 4).

The redundant part of the transverse colon and its mesentery, attributable to PDM, caused the twist.

Based on these findings, a diagnosis of recurrent transverse colon volvulus was established. Attempts at endoscopic detorsion were successful initially, but the patient experienced four subsequent volvulus episodes within a month. Consequently, the decision was made to proceed with laparoscopic partial resection of the transverse colon.

**Figure 5:**
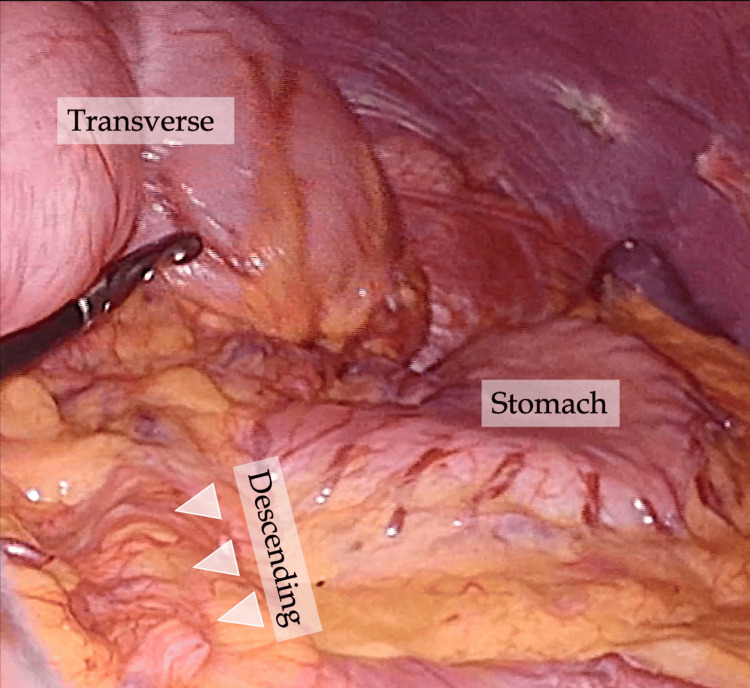
Operative findings Intraoperative observations reveal a dilated transverse colon, with the descending colon (arrowheads) positioned medially to the left renal hilum and adherent to the left wall of the abdominal aorta.

During surgery, the patient was positioned in the Trendelenburg position, and three ports were placed at the umbilicus and the right lower and lateral abdomen. Intraoperative observations revealed the descending colon positioned medially to the left renal hilum, adhering to the left wall of the abdominal aorta (Figure 5).

The transverse colon appeared dilated and redundant with a narrower mesentery. The part where colopexy was performed only left thin band strings, and the fixation to the abdominal wall came loose. The length of the PDM was not obvious because the descending colon was directly connected to the rectum. No sigmoid colon was observed. The PDM adhered to the retroperitoneum at the root of the volvulus (the top of the descending colon), but the rest of the descending colon did not adhere. Approximately 30 cm of the dilated transverse colon was resected and anastomosed using the functional-end-to-end method. Subsequent histopathological analysis confirmed the presence of a ganglionic segment in the transverse mesocolon, ruling out Hirschsprung disease. However, the patient developed anastomotic stenosis with leakage, necessitating a re-operation. Seven days after the second surgery, she was discharged without complications.

## Discussion

This case report delineates an unprecedented occurrence of recurrent transverse colon volvulus associated with PDM, marking the world's first documented instance of such an interplay. The case holds instructional significance for two primary reasons. First, PDM emerges as a potential causative factor for volvulus due to the redundant transverse colon. Second, the heightened risk of anastomotic failure in PDM cases is attributed to the shortened mesentery of the transverse colon, impacting vascular integrity.

Volvulus, characterized by the twisting of a colon loop around its mesentery, leads to obstruction and compromised vascular perfusion when the torsion surpasses 180 degrees [9]. While the exact pathophysiology remains unclear, anatomic predispositions, such as a lengthy redundant colon with a narrower mesenteric attachment, contribute to the condition. In the context of sigmoid volvulus, chronic fecal overloading from constipation may elongate and dilate the sigmoid colon, particularly in older, institutionalized individuals with neurologic or psychiatric conditions [10]. Colonic dysmotility in children with Hirschsprung disease is also recognized as a predisposing factor [11]. Our patient, lacking documented clinical or pathological risk factors for volvulus, suggests an alternative factor, possibly PDM, contributing to her symptoms. The midline shift of the descending colon in PDM renders the transverse colon and its mesentery redundant, creating an axis for torsion and subsequent volvulus.

PDM is a rare anatomic variant stemming from the failure of colonic mesentery fusion with the abdominal wall during gestation [12]. The descending mesocolon is normally fused to the posterior and lateral peritoneum by the end of the fifth month of gestation. The descending barely adhered dorsally to the retroperitoneum and shifts to a medial position in PDM, and the transverse and sigmoid colon can be redundant. Although some cases showed the total loss of the transverse colon and the longer sigmoid colon due to PDM [13], our case had the longer transverse colon and loss of the sigmoid colon. PDM is confirmed when the descending colon is located medially to the left renal hilum [4]. Although often asymptomatic, recent studies have identified specific complications associated with PDM, including primary colonic varices [14], sigmoid colon volvulus [15], prolonged operative time, anastomotic failure [6], and postoperative anastomotic stenosis in left-sided colon cancer [16]. The increased risk of anastomotic failure in PDM cases is linked to the incompletely expanded mesentery of the descending and sigmoid colon, resulting in a shorter length between the inferior mesenteric artery (IMA) and the marginal artery and colon. This contributes to a higher incidence of marginal vascular arch injuries and weaker blood supply at the proximal end of the. Despite our patient being an 18-year-old adolescent, the measured distances (21 mm and 24 mm) were shorter than reported averages (27 mm and 35 mm) [6], likely contributing to her postoperative stenosis, requiring reoperation. The potential use of intraoperative indocyanine green (ICG) to ensure adequate blood supply during the operation is emphasized. We should have utilized ICG during the initial operation to avoid her postoperative complication.

In the realm of sigmoid colon studies, endoscopic reduction of volvulus has shown success rates ranging from 75 to 95 percent [17]. However, recurrence rates remain high (up to 84 percent) following initial successful decompression [18]. Nonresectional surgical techniques, including detorsion with or without fixation (colopexy), and mesentery tailoring (mesopexy), are generally inferior to sigmoid resection in preventing recurrent volvulus [19]. In our case, given the frequent recurrence and post-colopexy leaving tissue string that put her at risk of potential strangulated bowel obstruction, a laparoscopic transverse colectomy was performed during the index admission.

Surgeons undertaking colectomy must possess a comprehensive understanding of the potential etiologies and risks associated with PDM. Familiarity with anatomic variations and anomalies proves crucial for delivering appropriate surgical treatment and evaluating preoperative complicative risks. Once confirmed, the surgical focus should include the meticulous isolation of mesenteric layers and the assessment of blood flow to the anastomosed colon, advocating for the use of intraoperative ICG.

## Conclusions

This report underscores a unique case of transverse colon volvulus attributed to PDM. While infrequent, the acknowledgment of PDM assumes paramount importance for surgeons. Overlooking such anomalies could precipitate unforeseen consequences, including recurrent volvulus and postoperative complications following colectomy. Surgeons are urged to maintain a heightened awareness of this anomaly, crucial for preemptively mitigating potential surgical risks.
